# Can Automated Vehicles Be Useful to Persons Living With Dementia? The Perspectives of Care Partners of People Living With Dementia

**DOI:** 10.1093/geront/gnab174

**Published:** 2021-11-19

**Authors:** Shabnam Haghzare, Ghazaleh Delfi, Elaine Stasiulis, Hodan Mohamud, Erica Dove, Mark J Rapoport, Gary Naglie, Alex Mihailidis, Jennifer L Campos

**Affiliations:** Institute of Biomedical Engineering, University of Toronto, Toronto, Ontario, Canada; KITE Research Institute, Toronto Rehabilitation Institute—University Health Network, Toronto, Ontario, Canada; KITE Research Institute, Toronto Rehabilitation Institute—University Health Network, Toronto, Ontario, Canada; Rotman Research Institute, Baycrest Health Sciences, Toronto, Ontario, Canada; Institute of Medical Science, University of Toronto, Toronto, Ontario, Canada; KITE Research Institute, Toronto Rehabilitation Institute—University Health Network, Toronto, Ontario, Canada; KITE Research Institute, Toronto Rehabilitation Institute—University Health Network, Toronto, Ontario, Canada; Department of Psychiatry, Sunnybrook Health Sciences Centre, Toronto, Ontario, Canada; Department of Psychiatry, University of Toronto, Toronto, Ontario, Canada; Department of Medicine and Rotman Research Institute, Baycrest Health Sciences, Toronto, Ontario, Canada; Department of Medicine and Institute of Health Policy, Management and Evaluation, University of Toronto, Toronto, Ontario, Canada; KITE Research Institute, Toronto Rehabilitation Institute—University Health Network, Toronto, Ontario, Canada; Department of Occupational Science & Occupational Therapy and Institute of Biomedical Engineering, University of Toronto, Toronto, Ontario, Canada; KITE Research Institute, Toronto Rehabilitation Institute—University Health Network, Toronto, Ontario, Canada; Department of Psychology, University of Toronto, Toronto, Ontario, Canada

**Keywords:** Assistive technologies, Care partners, Driving, Qualitative study, Semistructured interview

## Abstract

**Background and Objectives:**

Driving cessation is a complex challenge with significant emotional and health implications for people with dementia, which also affects their family care partners. Automated vehicles (AVs) could potentially be used to delay driving cessation and its adverse consequences for people with dementia and their care partners. Yet, no study to date has investigated whether care partners consider AVs to be potentially useful for people with dementia.

**Research Design and Methods:**

This mixed-methods study assessed the views of 20 former or current family care partners of people with dementia on AV use by people with dementia. Specifically, questionnaires and semistructured interviews were used to examine care partners’ acceptance of AV use by people with dementia and their views about the potential usefulness of AVs for people with dementia.

**Results:**

The results demonstrated that care partners identified possible benefits of AV use by people with dementia such as their anticipated higher social participation. However, care partners also voiced major concerns around AV use by people with dementia and reported significantly lower levels of trust in and perceived safety of AVs if used by the person with dementia in their care compared to themselves. Care partners’ concerns about AV use by people with dementia included concerns around the driving of people with dementia that AVs are not designed to address; concerns that are specific to AVs but are not relevant to the nonautomated driving of people with dementia; and concerns that arise from existing challenges around the nonautomated driving of people with dementia but may be exacerbated by AV use.

**Discussion and Implications:**

Findings from this study can inform future designs of AVs that are more accessible and useful for people with dementia.

## Background and Objectives

### Driving With Dementia and the Role of Care Partners

Symptoms of dementia, such as Alzheimer’s disease, can affect driving safety, leading to the eventual need for complete driving cessation for people with dementia. However, the rate of disease progression and associated driving decline varies greatly among individuals. Therefore, a diagnosis of dementia does not automatically lead to the immediate revocation of one’s driver’s license, recognizing that many people with early-stage dementia will still be able to drive safely for a period of time (Canadian Medical Association, 2019). On the other hand, people with dementia may lack insight regarding their own impairments (Starkstein et al., 2006) and as such may not use appropriate self-regulation strategies (Diller et al., 1999). This potential lack of insight is one of the reasons why driving and dementia pose unique challenges, requiring distinctive considerations that entail the involvement of family care partners in decisions around the driving of people with dementia. These challenges and considerations can be categorized into those that occur before and after complete driving cessation.

#### Predriving cessation

Prior to the complete driving cessation of people with dementia, the stages of driving decline have been described as consisting of an early stage with increasing concerns, which is sometimes followed by a crisis stage triggered by a particularly worrying event (e.g., near-crash; Liddle et al., 2013). Care partners are often actively involved with the decisions surrounding the driving activities of people with dementia and/or take initiative to involve health care professionals in these decisions (Adler et al., 1999). The negative emotional impact and the adverse health consequences of driving cessation for people with dementia (Sanford et al., 2020) complicate the decision regarding the appropriate timing of their driving cessation. Family care partners, who are often already facing emotional strains and demands placed on them by caring for someone with dementia (Ory et al., 2000), may avoid raising the topic of driving cessation with people with dementia in anticipation of negative emotional reactions (D’Ambrosio et al., 2009). However, driving cessation is an inevitable outcome for people with dementia and the timing of this decision should be made with careful consideration given that premature cessation can put people with dementia at risk of adverse health consequences sooner than necessary, and delayed cessation can compromise road safety and lead to motor vehicle injury or death.

The problem of determining the appropriate timing of license revocation remains a complex decision for physicians, who often report that they lack the training, tools, knowledge, and/or confidence to make an assessment of the fitness of people with dementia to drive (Jang et al., 2007; Rapoport et al., 2018). The sensitivity of this decision and the potential negative impact on the physician–patient relationship contributes to the physician’s reluctance to raise this topic with people with dementia (Andrew et al., 2015). This means that the onus often falls on the care partner to initiate conversations about or decide the timing of driving cessation for the person with dementia in their care.

#### Postdriving cessation

Complete driving cessation is often viewed by people with dementia as being disruptive to their independence and sense of identity (Sanford et al., 2019, 2020) and has negative health consequences, including increased depression and 3-year mortality rates (Chihuri et al., 2016), faster cognitive decline (Choi et al., 2014), and increased risk of institutionalization (Freeman et al., 2006). The driving cessation of people with dementia also affects care partners upon whom people with dementia often heavily depend for transportation (Taylor & Tripodes, 2001). Family care partners consistently report facing greater challenges associated with caregiving post- compared to predriving cessation (Connors et al., 2020; Seiler et al., 2012). Developing a range of alternative transportation options for people with dementia who no longer drive is important for mitigating the increasing challenges faced by care partners that can occur postdriving cessation, especially for people with dementia/care partners who live in rural areas with limited access to public transportation (Holden & Pusey, 2021; Taylor & Tripodes, 2001).

### Using Automated Vehicles as a Potential Alternative to Driving Cessation for People With Dementia

Automated vehicles (AVs) can perform, or assist with, some or all driving responsibilities (e.g., lane-keeping, speed control assistance, or both). AVs could, in theory, be used as an alternative to the nonautomated driving of people with dementia (Knoefel et al., 2019). As such, AVs can be viewed as assistive technologies that could help people with dementia drive safer and longer through two distinct features: (a) *assisting* people with dementia in performing some driving tasks, thereby prolonging their safe driving period (partially automated), or (b) *fully performing* all driving tasks for people with dementia and thereby relieving them from all driving responsibilities (fully automated). *Partially automated vehicles (PAVs)*, or Level 2 driving automation, can perform both steering and speed control, but the human driver is required to monitor all tasks at all times. *Fully automated vehicles (FAVs)*, or Level 5 driving automation, will be able to perform all driving tasks in all driving conditions, with no need for input from the human driver and potentially no means of allowing human input or intervention (SAE International, 2021). Whereas some PAVs are currently on the road, FAVs (the eventual targeted goal of the automotive industry) are currently undergoing development and testing.

Different types of AVs may hold varying levels of potential in assisting people with dementia to drive safer and/or longer. However, to the best of our knowledge, no studies to date have explored the potential usefulness of AVs in addressing the driving challenges faced by people with dementia. An initial way of examining this is to assess the views of family care partners of people with dementia. In this exploratory mixed-methods study, we conducted questionnaires and semistructured interviews with care partners of people with dementia to assess their perceptions of the potential usefulness of FAVs and PAVs for people with dementia in their care. This included, for example, caregivers’ trust in, perceived usefulness, and perceived safety of FAV and PAV use by people with dementia in their care.

## Research Design and Methods

### Participants

Twenty former or current care partners of people with dementia participated in the study. Participants were eligible to participate if they were fluent in English and self-identified as a family member or a friend who lives, or in the past has lived, with a person with dementia, or is/was centrally involved in providing and/or organizing unpaid care for a person with dementia. The people with dementia in the participants’ care are/were driving after receiving their diagnosis of dementia. The protocol was approved by the University of Toronto Research Ethics Board (#38808). A former care partner of a person with dementia was involved in the design process of the study to provide consultation on the study’s length, procedure, and instruments.

### Study Design and Setting

This study used questionnaires and semistructured interviews as the primary means of data collection to allow for an in-depth exploration of participants’ experiences and opinions. The study was conducted using a video-conferencing platform, with at least two members of the research team present. After obtaining consent, the interview questions and responses were audio-recorded and securely stored. In addition to being verbally asked the questions, the response options, where applicable, were shown to the participants on the screen.

### Instruments

#### History Questionnaire

The History Questionnaire (Supplementary Material A) was a brief questionnaire pertaining to participants’ demographic information, their relationship with the person with dementia, their involvement with the driving-related decisions of the person with dementia, and the diagnosis and driving history of the person with dementia. The section on driving history was adapted from Owsley et al. (1999).

#### AV Familiarity Questionnaire

The AV Familiarity Questionnaire (Supplementary Material B) included two 3-point Likert scale questions on the level of familiarity and experience with a current commercially available AV, Tesla Autopilot. Specifically, the questions asked, “How familiar are you with Tesla Autopilot?” and “How much experience do you have with Tesla Autopilot?” Participants were asked to rate their familiarity/experience with the Tesla Autopilot from zero (i.e., not familiar/no experience) to three (i.e., familiar/experienced).

#### AV Acceptance Questionnaire and Interview

The Acceptance Questionnaire and Interview (Supplementary Material C) aimed to capture care partners’ acceptance of PAVs/FAVs for use by themselves and for use by the person with dementia in their care. Items including trust in, perceived safety of, and intention to use AVs were adapted from the validated Autonomous Vehicle Acceptance Model Questionnaire (Hewitt et al., 2019) and summarized using three 4-point Likert scale questions. We selected three noted constructs because they best addressed the objectives of the study. In addition, for each of the three constructs included in our study, we chose only the one question that explicitly stated the name of the construct in the written question (i.e., Trust, Safety, Intention to Use). Furthermore, the response options were streamlined to a 4-point Likert scale instead of the 5-point scale based on the recommendation of our care partner consultant to avoid neutral responses. For each factor, participants were asked the questions twice consecutively, once as applied to PAV/FAV use by themselves and the other applied to PAV/FAV use by the person with dementia in their care. After each question, when applicable, participants were asked to provide their reasons if and/or why their answers differed for PAV/FAV use for themselves compared to the person with dementia in their care. The questions were repeated as related to PAVs and FAVs separately, but in each case, the questions were framed differently to reflect PAV or FAV use.

#### AV Usefulness Questionnaire and Interview

The AV Usefulness Questionnaire and Interview (Supplementary Material D) was created to capture care partners’ perceived usefulness of PAVs/FAVs in mitigating the potential driving challenges of people with dementia, with two sections thematically related to (a) challenging driving *conditions* (e.g., nighttime, heavy traffic) and (b) challenging driving *tasks* (e.g., left turns, backing up the car). The conditions/tasks were chosen to represent situations commonly avoided by older adults (Tuokko et al., 2014). Participants were asked whether, in the context of nonautomated driving, they would “discourage the person with dementia in their care from driving” in a particular driving condition or performing a particular driving task. If they responded “yes,” the subsequent question asked whether they “would still discourage them if they use a PAV/FAV” for each condition and task. Specific to each driving condition/task, the participants were asked to reflect on the reasons why they would encourage or discourage the person with dementia in their care from using a PAV/FAV. Two versions of the AV Usefulness Interview were implemented and used, as applicable to PAVs and FAVs.

#### FAV Trip Cognitive Walkthrough Interview

A cognitive walkthrough is a task-specific usability inspection method, in which the participant is guided through a sequence of actions, for instance using a storyline, as a formalized way of imagining the users’ thoughts and actions (Mahatody et al., 2010). The objective of the FAV Trip Cognitive Walkthrough interview was to allow participants to reflect on the usefulness of FAVs to address the potential challenges that people with dementia may face in the entirety of a trip, even when the FAV is fully performing all driving tasks for the person with dementia. This included aspects of the trip beyond simply the act of driving, such as leaving their residence to get to the car, traveling to the intended destination, engaging in the intended activity, and returning home. Two brief scenarios were described to the participants, in which they were asked to mentally walk through the actions required for the person with dementia to take a trip to a grocery store using a FAV. In both scenarios, participants were asked to reflect on the potential obstacles that the person with dementia may face from leaving their home, to using the FAV, finding the grocery store, and finding their way back home. The second scenario differed only in that the participants were asked to reflect on the response of the person with dementia to a situation in which the automated system disengages due to a system failure.

### Procedure

In order to ensure that all participants were aware of the capabilities of the two types of AVs, AV functionality briefings as plain language summaries were provided both verbally and shown on the screen to describe the automation functionality and the driver’s responsibilities in PAVs and FAVs. These briefings were provided immediately before the questionnaires and interviews about PAVs and FAVs, respectively. These descriptions included tables adapted from Seppelt et al. (2018), showing the allocation of driving responsibilities between the PAV and FAV.

**Figure 1.**
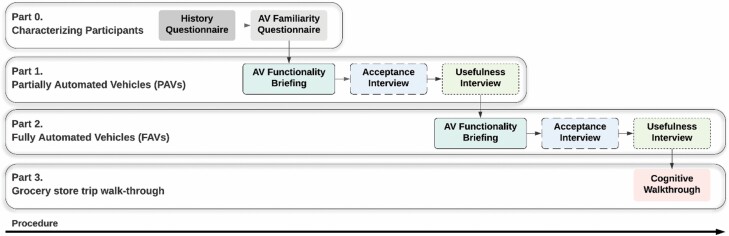
A schematic of the procedure of the study. AV = automated vehicle.

As shown in Figure 1, after obtaining informed consent, participants completed the History Questionnaire and AV Familiarity Questionnaire. An AV functionality briefing was provided, followed by the interview components relating to Usefulness and Acceptance. This was done first for PAVs and subsequently for FAVs. The Cognitive Walkthrough interview was the final interview administered. In cases where the individual in their care had stopped driving, participants were instructed to base their answers on the time frame immediately before the time when the person with dementia stopped driving. The length of the interviews varied among participants from 40 min to 2 h.

### Data Analysis

To qualitatively assess the participants’ responses to the open-ended questions, audio recordings of the semi-structured interview sessions were manually transcribed verbatim and subsequently reviewed for accuracy by two members of the research team. An inductive thematic analysis was conducted (Braun & Clarke, 2006), and the text data were assessed and coded by two members of the research team using NVivo 12 (S. Haghzare and G. Delfi). The data relevant to each code were collated separately by each of the two study members for the entire data set. In an iterative process, the initial codes were searched, and the two sets of codes generated by the two study members were compared to create a coding framework and maps. Consequently, the transcripts were re-coded using the coding framework, and the codes were grouped into recurring patterns (or “themes”). Themes were identified and further categorized into higher-level themes and a thematic map was created. The procedural rigor was maintained by ongoing documentation of field notes, transcripts, codes, definitions, and mappings. The discrepancies were resolved by discussion between the two study members. The participants’ responses to the questionnaires were analyzed using nonparametric statistical analyses as was appropriate given the small sample size and the ordinal data collected using the questionnaires. The specific types of statistical analyses are described where applicable in the *Results* section.

## Results

### Results of the Questionnaires

#### Participant characteristics

Table 1 describes the study participants separated by sex. The study included 20 former or current care partners of people with dementia (age range = 33–79 years). The participants’ relationship to the person with dementia, in order of frequency, ranged from child, spouse, and grandchild, to neighbor or friend. Participants identified themselves as being either extremely (70%) or somewhat (30%) involved in decisions pertaining to the driving of the person with dementia. Based on the AV Familiarity Questionnaire, 90% of participants had no prior experience with Tesla Autopilot as a current commercially available AV, but 65% reported some level of familiarity.

**Table 1.** Factors Characterizing Participants Separated by Sex

Variable		Female (*n* = 11)	Male (*n* = 9)	All participants (*n* = 20)
**Characterizing participants (i.e., care partners)**				
*Demographics*				
Age	*M* (*SD*)	62.36 (14.61)	61.44 (12.51)	61.95 (13.35)
Years of driving experience	*M* (*SD*)	41.45 (15.49)	41.33 (15.72)	41.40 (15.17)
*Self-reported prior knowledge of AVs*				
Familiarity with Tesla	*Not familiar*			35%
	*Slightly familiar*			50%
	*Familiar*			15%
Experience with Tesla	*No experience*			90%
	*Some experience*			5%
	*Experienced*			5%
**Characterizing the person with dementia in the participants’ care**				
*Relationship to the person with dementia*				
Relationship	*Child*	30%	35%	65%
	*Spouse*	15%	5%	20%
	*Grandchild*	5%	—	5%
	*Neighbor*	5%	—	5%
	*Friend*	—	5%	5%
Self-reported involvement driving-related decisions of the person with dementia	*Extremely*			70%
	*Somewhat*			30%
	*Not at all*			—
*Diagnosis of the person with dementia*				
Dementia type	*Alzheimer’s*			40%
	*Unknown*			20%
	*Vascular*			5%
	*Lewy body*			5%
	*Mixed*			5%
	*Frontotemporal*			5%
	*Parkinson’s*			5%
Dementia stage when stopped driving	*Mild*			50%
	*Moderate*			20%
	*Unknown*			15%
	*Moderate to severe*			10%
	*Mild to moderate*			5%

*Notes:* AV = automated vehicle. Levels are organized from high to low frequency.

#### Care partners’ perceptions of PAV/FAV use by themselves or by the person with dementia in their care

**Figure 2.**
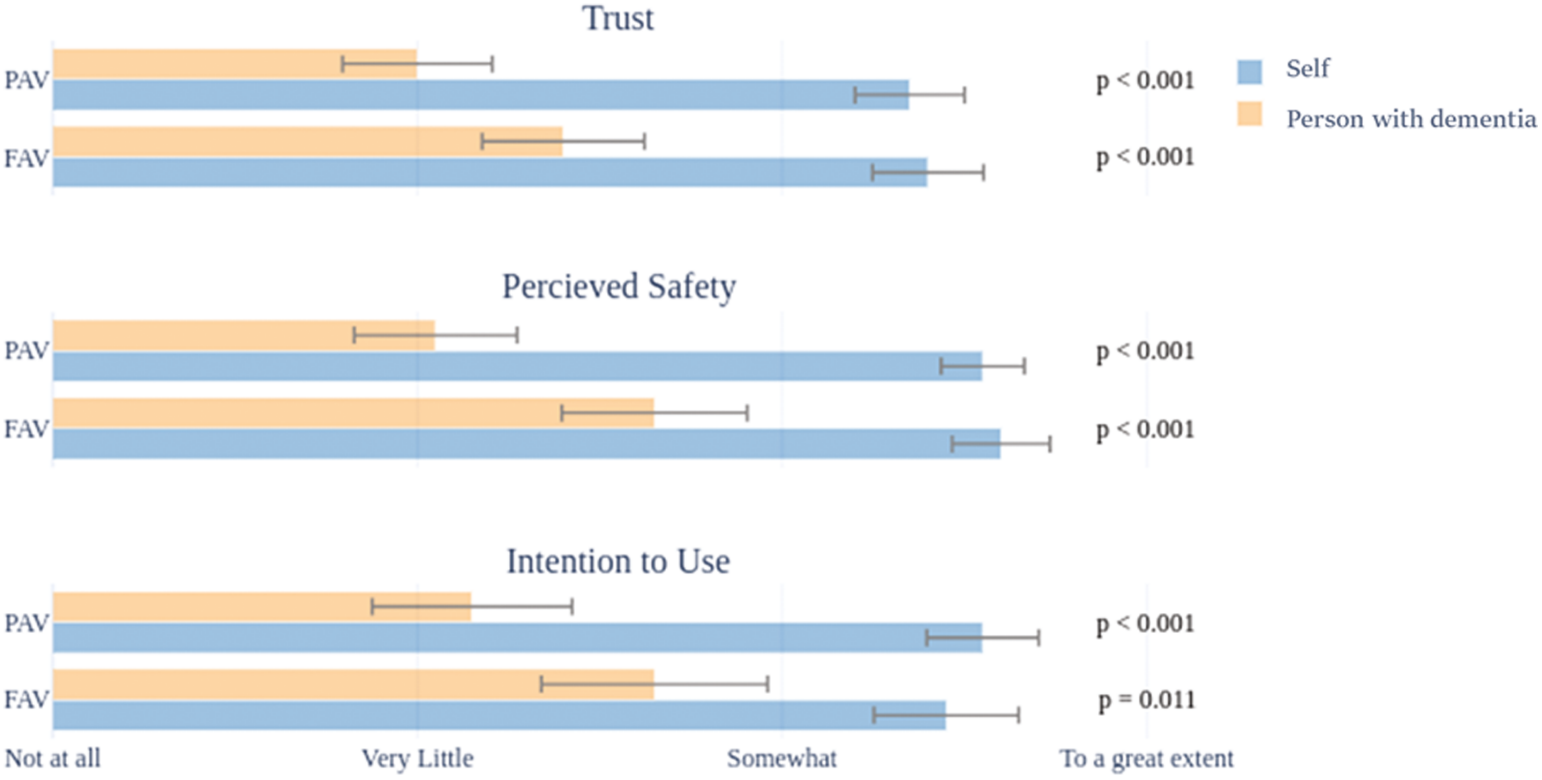
Care partners ranking of PAV and FAV use by themselves (self) and the person with dementia in their care for three factors of trust, perceived safety, and intention to use. *Note:* PAV = partially automated vehicle; FAV = fully automated vehicle. The *p* values are the results of Wilcoxon signed-rank tests conducted to examine the difference in care partners’ perceptions of PAV/FAV if used by themselves compared to the person with dementia (self vs. person with dementia). Mean and standard deviations of the ratings are shown.

Figure 2 shows the comparison of the participant ratings of trust in, perceived safety of, and intention to use PAVs and FAVs if used by themselves versus the person with dementia. To examine the difference in care partners’ perceptions of PAV/FAV if used by themselves compared to the person with dementia in their care (self vs. person with dementia), six separate Wilcoxon signed-rank tests were conducted for each factor (i.e., trust, perceived safety, and intention to use) and for each AV type (PAV vs. FAV). Based on an adjusted significance level of 0.008 using the Bonferroni correction for multiple comparisons, all except one comparison between intention to use FAVs for self versus person with dementia were significantly different, indicating a more positive perception of FAV or PAV use by care partners themselves compared to use by people with dementia (Figure 2).

To examine the effects of AV type (PAV vs. FAV) on care partners’ perception of AV use, six separate Wilcoxon signed-rank tests were conducted for each factor (trust, safety, intention to use) and for each group (self, people with dementia). The results indicated no significant effect of AV type on any of the factors, for use by self or for use by people with dementia (all *p* values were higher than .5).

#### Effects of driving conditions/tasks on care partners’ perceptions of the use of PAV/FAV by people with dementia

**Figure 3.**
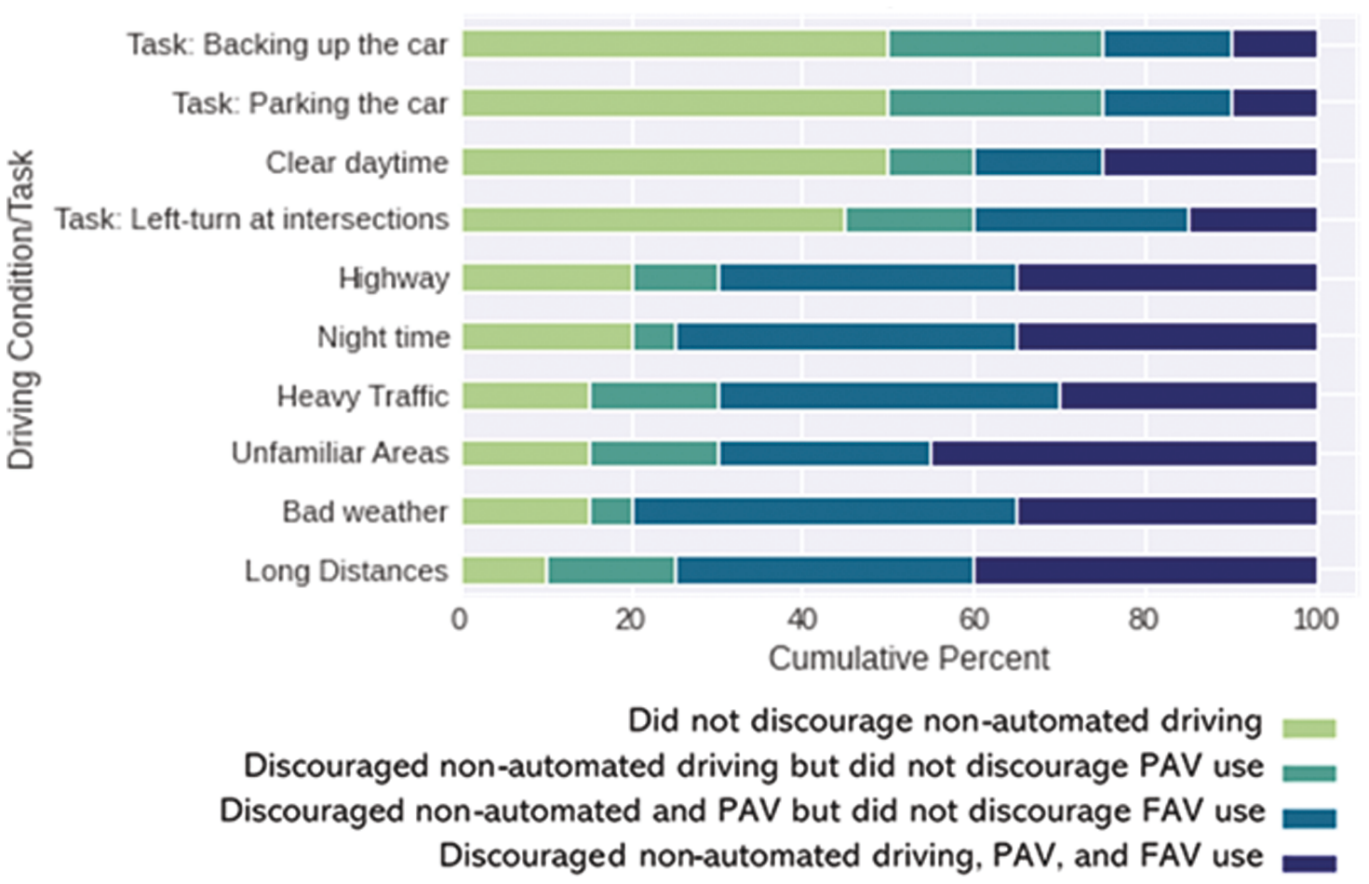
A stacked bar chart of the percentage of care partners’ perception of the driving of people with dementia with nonautomated vehicles, PAVs, and FAVs as separated by driving conditions/tasks. PAV = partially automated vehicle; FAV = fully automated vehicle.

To assess the effects of driving conditions/tasks on care partners’ view of FAV and PAV use by people with dementia under the 10 selected driving conditions/tasks listed in Figure 3 (dichotomized variable), we conducted two separate Cochran’s *Q* tests (Cochran, 1950). The results indicated significant effects of driving condition/task on care partners’ reported intention to encourage/discourage the use of both PAVs (χ ^2^(9) = 51.72, *p* < .001) and FAVs (χ ^2^(9) = 30.21, *p* < .001) by the people with dementia in their care. Follow-up McNemar’s tests between all driving condition/task pairs indicated that a significantly higher proportion of care partners would discourage PAV use by people with dementia in adverse weather conditions compared to PAV use to back up the car (χ ^2^(1) = 11.00, *p* < .001) and park the car (χ ^2^(1) = 11.00, *p* < .001). In contrast, a lower proportion of care partners would discourage FAV use by people with dementia during nighttime compared to FAV use to back up the car (χ ^2^(1) = 8.07, *p* = .005) and park the car (χ ^2^(1) = 8.07, *p* = .005).

### Results of the Interviews

#### Care partners’ perceived benefits and shortcomings of AV use by people with dementia

The extracted themes were categorized into care partners’ perceived *benefits* (Table 2) and *shortcomings* (Table 3) of AV use by people with dementia. The shortcomings were categorized into three major themes: (a) *emerging concerns* about the driving of people with dementia that could be introduced if they use AV technology instead of non-AVs, (b) *unresolved concerns* about the driving of people with dementia that may persist after AV use and are not addressed by AV technology, and (c) *exacerbating concerns* about the nonautomated driving of people with dementia that may be further intensified by AV use. Where applicable, the differences in themes identified for PAV versus FAV use are noted in tables. Otherwise, the themes identified for FAVs and PAVs were the same. All the identified themes are based on the care partners’ view of the independent use of PAVs/FAVs by people with dementia in the absence of their care partner(s).

**Table 2.** Categorization of Major Themes Identified by Care Partners as the Perceived Benefits of AV Use by People With Dementia

Theme	Examples
B.1. Convenience for everyone in the circle of care	B.1.1. AVs providing freedom to care partners who take on the responsibility of driving the person with dementia
B.2. Benefits to the life participation and independence of people with dementia	B.2.1. Usefulness of AVs in enabling the social participation of people with dementia (e.g., access to social activities or other occasions that do not otherwise get prioritized compared to, for example, medical appointments)
	B.2.2. Usefulness of AVs in enabling the independence of people with dementia
	B.2.3. AVs could be better than the current alternatives to driving (e.g., public transportation)

*Notes:* AV = automated vehicle. B stands for themes under “benefits.”

**Table 3.** Categorization of Major Themes in Care Partners’ Perceived Shortcomings of PAV Use by People With Dementia

Category	Theme	Examples
Emerging concerns	S.1 AV compatibility with the abilities/preferences of people with dementia	S.1.1 Possible distress/agitation of people with dementia in AVs • Concerns more pronounced in FAVs than PAVs
		S.1.2 Potential challenges of people with dementia performing tasks required by AVs • *Specific to FAVs:* Response to system failures, negotiating pick-up/drop-off locations, or changing destinations mid-way • *Specific to PAVs:* Performing the driving responsibilities even with the assistance of the PAV
		S.1.3 Potential lack of willingness of people with dementia to use AVs
Unresolved concerns	S.2 The nondriving tasks required to use the vehicle	S.2.1 AVs still requiring people with dementia to independently get in the car, fasten seatbelt, etc.
	S.3 The tasks required for taking a trip, before and/or after driving	S.3.1 Potential lack of ability of people with dementia to initiate or complete a trip, for instance, locating the destination (e.g., doctor’s office) or finding keys to the vehicle
	S.4 The decision of driving cessation	S.4.1 Potential lack of awareness about the declines in the driving performance of people with dementia in cases of fast progression
	S.5 The emotional distress of ceasing nonautomated driving	S.5.1 AVs not providing the same sense of freedom as nonautomated driving
Exacerbating concerns	S.6 Potential wandering	S.6.1 AVs allowing more dangerous wandering behavior compared to walking, nonautomated driving, or other means of transportation
	S.7 Potential driving skill loss/decay	S.7.1 AVs accelerating the driving skill loss in people with dementia because of the lack of driving practice enabled by partial or complete performance of driving tasks by PAVs/FAVs

*Notes:* AV = automated vehicle; FAV = fully automated vehicle; PAV = partially automated vehicle. S stands for themes under “shortcomings.”

#### Care partners’ perceived benefits of AV use by people with dementia

Participants described several potential benefits to both the person with dementia and caregiver(s) if PAVs/FAVs could augment and/or restore the safe driving ability of the person with dementia. A major theme identified in the anticipated benefits of PAVs/FAVs was enabling the continued independence of people with dementia, especially as related to participation in social activities, which would have otherwise not been prioritized. For example, “she [the person with dementia] could go to see her friends, she could go out for an evening and since they were not accessible by transit, she had to drive … she needed those outings, and I frankly wasn’t in a position to drive her all the time either.” This anticipated independence of people with dementia enabled by AVs was perceived to benefit the entire circle of care because the responsibility of caring for a person with dementia may be shared among multiple family members/friends. For example, as expressed by:

It’s not just more freedom for me, it’s more freedom for everyone in the care circle.

However, the care partners’ anticipated benefits of AV use by people with dementia often coincided with an expressed understanding that they would have to take a leap of trust in using AVs and assume that they “would trust that they [AVs] were doing everything they [manufacturing companies] said they [AVs] would do.” For example:

I would trust the system to work for her [the person with dementia] so that she could continue to have her independence. I’d have to do that because I’d want her to have her independence, so I think that I would have to make myself. Like it’s just like now, where we have workers coming in right, and you know in this COVID situation, I have to trust that the workers are keeping themselves safe with my mom.

Similarly, in other instances, this trust leap was conditioned on the availability of a perfectly safe AV on the market: “I would trust that they would not put it on the market unless it was performing perfectly without any question of danger or going the wrong way or braking too soon or not braking.” Some care partners additionally noted that the AV benefits for the person with dementia in their care may drive them to trust commercially available AVs: “I would trust the system to work for her [the person with dementia] so that she could continue to have her independence. I’d have to do that because I’d want her to have her independence.”

##### Category 1 of AV shortcoming: care partners’ emerging concerns around AV use by people with dementia.

The first and most mentioned category of care partners’ concerns identified was *emerging concerns* around the driving of people with dementia that do not exist for their nonautomated driving. These new concerns may be introduced, for example, because of the lack of AV compatibility with the capabilities and preferences of people with dementia. Three main themes were identified under this category. First, care partners indicated that there is a possibility that the person with dementia may become distressed or experience agitation in the AVs, especially in FAVs. For instance, there was expressed concern that “[the person with dementia] would not know where he is going and that will cause him a lot of distress,” or the “[possible] lack of understanding [of the FAV functionality] would cause [the person with dementia] quite a bit of stress.” The unfamiliarity of the person with dementia with the concept of a driverless car was attributed to their possible stress in an FAV: “I really think that she’d have trouble being in a car without a driver, that would just freak her [the person with dementia] right out.” This unfamiliarity with AVs was also identified as a disturbance to the consistency and stability required by people with dementia:

He [the person with dementia] always requires a very stable and consistent environment. Quite often when I would be taking him to appointments or for rehab it would be always during the daytime and would always be the same route. So, there was a certain amount of consistency to it. So, to change anything, for example to go into a highway, would cause him a lot of confusion and probably a lot of distress. The same would apply to the fully automated vehicle even though the driving is being done by the car, he wouldn’t understand.

Second, care partners raised concerns about the ability of a person with dementia to perform tasks that may potentially be required of the human driver in the PAVs and FAVs. In PAVs, care partners voiced concerns about the ability of the person with dementia to perform the driving tasks, even with the PAV’s assistance because, “the aspects of driving that the PAV does not claim to cover are obviously therefore covered by the driver. And her [the person with dementia] executive functions were such that I regarded her and as did her physician as being untrustworthy, you know on the road to drive a vehicle. So, even a PAV would be better than not having it, but it wouldn’t be sufficient to make it safe for her to drive.”

In FAVs, care partners raised concerns about the abilities of people with dementia to perform tasks other than driving that are introduced by the FAVs. One such concern was pertaining to negotiating pick-up and drop-off locations where, “If they [people with dementia] have to program the destination, they aren’t able to do that unless the person with them in the car would do it.” Similarly, care partners raised concerns about the possibility of errors in negotiating the destination/drop-off location, “what if they [the person with dementia] accidentally inputted a different place and the car’s already going there and the person with dementia doesn’t realize that they are going to the wrong destination?” Additionally, concerns were also raised about the FAV drop-off process: “when he [the person with dementia] leaves the car, he probably would never find it,” or questioning whether the drop-off process is compatible with the abilities of people with dementia, “What happens just before the car moves away and when it gets to the destination? You cannot just let the person [with dementia] out of a car.”

In addition, despite the instructions in the FAV Functionality Briefing material where FAVs were defined as being able to perform all driving tasks at all times, care partners consistently reported concerns about the abilities of people with dementia to respond appropriately to “system failures” because “something could always go wrong” and “computers are not infallible.” For instance, one participant stated that, “I always think about system failure …. There is no way she [the person with dementia] could have responded appropriately or take over the responsibility.”

During the final walkthrough interviews, where the possibility of the people with dementia having to take over driving from the AV was intentionally introduced, care partners reflected on the reasoning behind their concern of the potential challenges of people with dementia with performing this task. Some care partners attributed the potential lack of ability of people with dementia to take over driving to “a matter of training.” In other words, care partners identified training as a measure to close the gap between the abilities of people with dementia and requirements of a system failure: “When they [people with dementia] get this fully automated vehicle, they [people with dementia] should be trained in these kinds of situations [system failures], so that it can be simulated that it suddenly disengages, and they [people with dementia] have to take over.” However, some noted that they have doubts in the effectiveness of training when dementia progresses because “training is learning. I think with her dementia, it’s very hard to take in that new information and retain that information. There would be a lot of repeating, but I am not 100% sure it would actually sink in.” Correspondingly, some other care partners noted that, in order for training to be effective, “training should have been before their dementia.” Or similarly, an exposure to AVs in the early stages of dementia was proposed as a solution to alleviate the potential distress of people with dementia in AVs, “if he [the person with dementia] is getting used to driving this kind of vehicle when he already started developing dementia it’s a completely different situation.”

The care partners identified AV design features as another contributing factor to the potential challenges of people with dementia in taking over driving control in case of failures, including “clear instructions” and to also “stop gradually” or “signal to the caregiver or someone else who is tracking this car and tell them that this [system failure] is happening.” This is because “they [the person with dementia] may not even realize the automation has disengaged.”

The third major theme in the category of emerging concerns was care partners’ anticipation of the reluctance of people with dementia to use PAVs or FAVs because, for instance, a care partner “noticed [that] she [the person with dementia] resists a lot of new things, new ideas, new suggestions,” and particularly in case of FAVs, that would be “too drastic a change from [what] she [the person with dementia] is used to.” This reluctance was voiced more consistently in the case of FAVs, which, compared to PAVs, “would have just been a bigger sort of technological leap. Because I literally would have to present it to her as think of it as ‘an automatic taxi’ as opposed to ‘you are going to take your car out’.” Another identified barrier to the use of PAVs/FAVs by people with dementia was the potential price of AVs because one can “take a lot of taxi rides for 80 thousand dollars [the care-giver’s estimated price of a PAV/FAV].”

**Figure 4.**
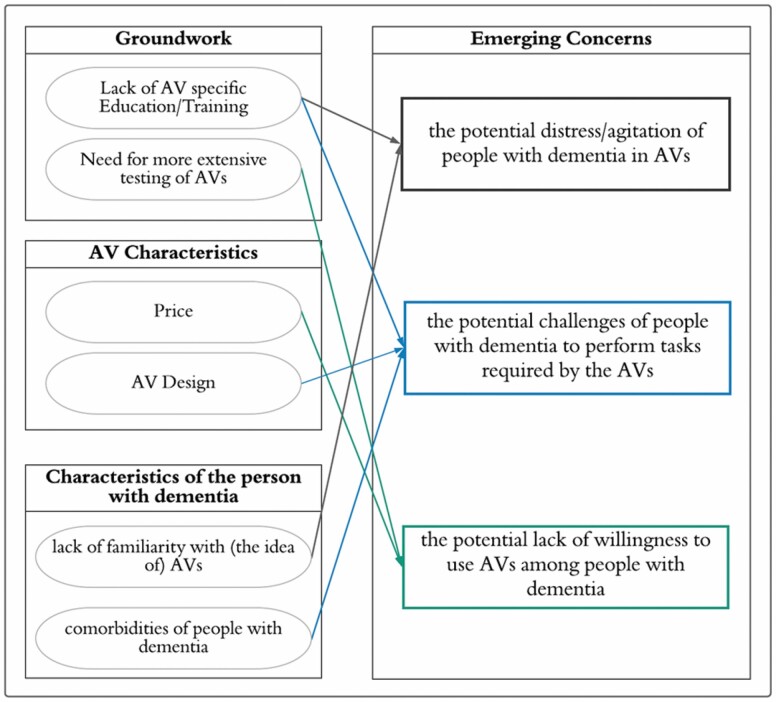
A thematic map of the reasons identified by care partners for their emerging concerns about the AV use of people with dementia, which are categorized into groundwork, AV characteristics, and characteristics of people with dementia. The identified reasons under each category are connected with an arrow to their corresponding concern. AV = automated vehicle.

To describe the reasoning of care partners behind their *emerging concerns*, the interview text categorized as *emerging concerns* was grouped into groundwork, AV characteristics, and the characteristics of the person with dementia as shown in the thematic map in Figure 4.

##### Category 2 of AV shortcoming: care partners’ unresolved concerns around AV use by people with dementia.

Another category of themes identified as AV shortcomings were the care partners’ concerns around the driving of people with dementia that are not addressed by AVs, for instance, care partners’ concerns about the ability of the person with dementia to perform other tasks in the car:

Sometimes it’s a struggle to put on the seat belt and lock the door and a partially automated vehicle would solve none of those, so I mean it’s a non-starter.

Similarly, recognizing that “it is not just about driving,” care partners had concerns about “all the stuff before and after that is involved in a journey,” that is, the tasks that people with dementia need to complete before getting in the car or afterward. For instance, when a person with dementia “can’t find his keys.”

The third identified theme was related to care partners’ concerns about the applicability of AVs to help avoid the early signs of driving decline in dementia. This is because even if AV use replaces the driving cessation of people with dementia, the appropriate timing of the transition from nonautomated driving to driving cessation/automated driving may not be apparent to care partners because neither the person with dementia nor the care partners were aware of the initial driving declines of the person with dementia: “[the person with dementia] would still be driving and comes home. We didn’t know. We thought everything was okay until we saw the side mirror hanging and this person [the person with dementia] doesn’t even realize.”

The fourth theme under the category of unresolved concerns was the AV’s limitation in addressing the adverse emotional implications of ceasing nonautomated driving for people with dementia because of uncertainties as to whether AVs will give people with dementia “a sense of freedom.” Similarly, care partners noted that the task of driving itself may be associated with this sense of freedom or being “in control.”

I don’t know if for someone like him [the person with dementia] he would miss driving himself, I think that would be missing cause his whole life he’s driving and now he’s just sitting there, and I think that would affect him psychologically a bit.

As a suggestion to alleviate AV’s potential limitation to help with the emotional implications of ceasing nonautomated driving, one care partner suggested: “if the apparatus was in the vehicle [FAV] just to give him [the person with dementia] a sensation of him driving the vehicle even if he’s not, and they’re non-functional—just to have them physically so he’d feel like he’s in control.”

##### Category 3 of AV shortcoming: care partners’ exacerbated concerns around AV use by people with dementia.

The third category of anticipated shortcomings of AV use for people with dementia was concerns that currently exist around the nonautomated driving of people with dementia that may be exacerbated by the AV use of people with dementia. For instance, a consistently voiced concern was the ability of AVs to enable riskier wandering behavior as AVs posed “the tremendous danger that she [the person with dementia] could end up in, being in any place in North America, because she put the wrong coordinates into the GPS.”

Another exacerbated concern by AVs was related to the identified potential for AV use to accelerate the declines in the driving performance of people with dementia:

Once lost a given skill, such as her ability to play solitaire with cards or certain other card games, she [the person with dementia] couldn’t get it back. Even though, once she starts relying on this system, the FAV, she would very quickly get used to it, get acclimatized to it, and like it and I would worry she would very quickly lose the ability to hop back in the driver’s seat.

## Discussion and Implications

### Care Partners’ Perceptions of AV Use by People With Dementia

For both PAVs and FAVs, care partners reported significantly higher levels of trust in and perceived safety of PAV/FAV use by themselves compared to use by people with dementia, with significant effects of driving condition on care partners’ views on PAV and FAV use by people with dementia. The between-AV comparisons revealed no significant effect of AV type (PAV vs. FAV) on care partners’ views of AV use by people with dementia. The themes identified in the interviews were first categorized into care partners’ perceived benefits and shortcomings of AV use by people with dementia. Notably, in terms of the benefits, care partners identified that AVs could enable the independence and life participation of people with dementia while mitigating the need for support from the “entire circle of care.” However, care partners often prefaced the identified benefits of AV use by people with dementia on the expectation that they will “have to” trust the safety of the AVs that will eventually be on the market. This trust leap, which is derived by the anticipated benefits of AVs for people with dementia, reinforces the importance of addressing the current gaps between the marketing presentations of AVs and their actual capabilities (Dixon, 2020). Misleading marketing claims about AVs can be especially harmful to those who see a significant benefit in using AVs in enhancing the quality of life of themselves/family members. False claims regarding the capabilities of the AV can, in turn, convince some cohorts, including people with dementia, to use AVs without having an accurate understanding of the AV’s functionality. This type of miscalibrated mental model of AVs can lead to AV misuse, which poses a significant safety risk (Parasuraman & Riley, 1997), especially given the minimal likelihood that the AVs are tested on cohorts with impaired driving abilities before commercial release.

The care partners also identified concerns around the use of AVs by people with dementia, which were grouped into three categories. The first and most mentioned category was *emerging concerns* about the driving of people with dementia that would only become relevant if people with dementia use AVs and are currently not relevant in the context of their nonautomated driving. These concerns included the possible distress of people with dementia in AVs (especially FAVs), the potential lack of willingness of people with dementia to use AVs, and the potential challenges of people with dementia in negotiating pick-up/drop-off locations and/or responding to system failures. Care partners attributed their emerging concerns to the lack of training of people with dementia with AVs, lack of extensive testing of AVs, AV price and design, and/or a lack of a-priori familiarity with AVs among people with dementia. Notably, care partners identified training as a potential means of reducing the distress of people with dementia during AV driving and a way to enhance the abilities of people with dementia to perform the tasks required by AVs (e.g., responding to failures and/or negotiating pick-up/drop-off locations). The AV training gap identified by the care partners for people with dementia is part of a larger insufficiency in effective AV training practices that are needed across a wide range of drivers (DeGuzman & Donmez, 2021). However, as highlighted by the caregivers, it is not yet clear whether it would be possible to effectively train people with dementia to use AVs safely. Future studies should assess whether it is possible to train people with dementia at various stages of dementia to use AVs safely. If so, future studies should further assess how AV training could be adapted for the effective training of populations with unique training requirements, such as people with dementia.

The second category of shortcomings identified by care partners were *unresolved concerns* around the driving of people with dementia that may persist after AV use and that are not addressed by AV technology. These shortcomings are mostly related to concerns with the abilities of people with dementia to perform nondriving tasks during an AV trip (e.g., fasten seatbelt, get in the car). These concerns pose a major challenge to the effective use of AVs by people with dementia and should, therefore, increasingly be the focus of future research if the benefits of AV use for people with dementia are to be realized. Similarly, future research should further assess the implications of the third category of shortcomings identified by care partners on the use of AVs by people with dementia which include exacerbating concerns about wandering behavior and driving skill decline of people with dementia.

The shortcomings and benefits of AV use by people with dementia as identified by their care partners highlight the potential for current and future AV systems to help people with dementia maintain their driving safety, but only if AVs are designed to be compatible with the abilities and preferences of people with dementia, and if people with dementia can use them appropriately.

### Study Limitations

As an exploratory study, a limitation of this work is that care partners were not strategically selected based on the type and stage of the diagnosis of the persons with dementia in their care, and they were not required to have been responsible for driving-related decisions of the persons with dementia. Different types of dementia are often associated with different symptoms (e.g., Lewy body dementia may involve motor symptoms, hallucinations, and fluctuations in the level of alertness), and later stages of dementia are associated with more severe cognitive impairment and functional impairment than earlier stages. As such, the stage and/or type of dementia can potentially influence the use of AVs by people with dementia, and thereby, influence care partners’ perception of AV use by people with dementia. Another related limitation of the study is that the care partners were asked to base their answers on the time frame immediately before the persons with dementia stopped driving completely. While only one care partner was currently caring for a person with dementia going through that transition, the remaining participants had to reflect retrospectively up to 10 years in the past, which could have implications regarding the answers they provided. In addition, while participants received a briefing on the drivers’ responsibilities in PAVs and FAVs, the majority (95%) lacked experience with currently available commercial AVs that could inform their judgment. Furthermore, most instruments were either adaptations and/or combinations of other validated questionnaires or were curated for the specific purpose of this study. As such, psychometric properties of the used instruments need to be further assessed.

### Future Work

To address the limitations of the current study, future studies should consider assessing a larger sample of care partners on their views on the use of AV by people with dementia. Additionally, when possible, the participants should strategically be selected among care partners of persons with dementia who are current drivers, or going through the transition to nondriving, and/or across individuals who have different types and/or severity levels of dementia. Additionally, future studies should investigate care partners’ acceptance of AV use by people with dementia after providing care partners with the experience of using AVs.

## Conclusions

Care partners saw significant potential benefits of AV use by people with dementia for both the person with dementia and the entire circle of care, such as enabling the social participation of people with dementia and increased freedom for care partners. However, the care partners reported significantly lower levels of trust in and lower perceived safety of AV use by people with dementia compared to their own AV use. Three categories of emerging, unresolved, and exacerbated care partners’ concerns around the use of AVs by people with dementia were identified. These concerns present a challenge to the anticipated potential for AV use to prolong the safe driving of people with dementia. The AV shortcomings identified by the care partners included potential lack of AV compatibility with the abilities and preferences of people with dementia and the potential challenges of people with dementia in navigating tasks required to complete a trip before and after driving. Most identified shortcomings stem from the fact that AVs, in their current state, are not designed to be an assistive technology and specifically are not designed for people with dementia. Ultimately, such human factors considerations of AV use will be key in adapting them to become an assistive technology and in developing guidelines and policies around the safe use of AVs by people with dementia. As such, the identified shortcomings can help inform the objectives of future AV designs and intelligent in-vehicle systems that are specifically designed for people with dementia, thereby potentially prolonging their safe and independent road mobility.

## Supplementary Material

gnab174_suppl_Supplementary_Material
