# Supplementary material for: Can Automated Vehicles Be Useful to Persons Living With Dementia? The Perspectives of Care Partners of People Living With Dementia
Source: Gerontologist. 2021 Nov 19;62(7):1050–62. doi: 10.1093/geront/gnab174 (PMC9372895; doi:10.1093/geront/gnab174)
Supplement: gnab174_suppl_Supplementary_Material [file gnab174_suppl_supplementary_material.docx]

# Online Supplementary Material

## Supplementary Material A. History Questionnaire

We are interested in your personal history because it may help us to better understand the results of our study. Your answers to a few short questions will aid us in this effort. All answers will be kept strictly confidential. Thank you for your help.

**Demographics:**

1. Age: _____
2. Sex: *(circle response)*
3. Male
4. Female
5. Living Arrangement: *(circle response)*
6. With household (family, souse or significant other, friends, roommate)
7. Living alone
8. Other, please specify: _______________
9. Place of birth: ________________________________

**Driving History:**

1. Do you have a valid driver’s license? YES/NO
2. How many years of driving experience do you approximately have? __________________________
3. How often do you approximately drive? *(circle response)*
   1. everyday
   2. sometimes
   3. never

**Person In Your Care**

**Diagnosis of the person with dementia in your care:**

1. What is/was their diagnosis of dementia [around the time when they were still driving]?

Stage: (1) mild, (2) moderate, (3) Severe, (4) N/A, (5) Unknown

Type: __________

1. What year they diagnosed with dementia? ___________

**Relationship to the person with dementia in your care**

1. What is your relationship with the person with dementia in your care? __________
2. To what extent are/were you involved with the decisions regarding the driving of the person with dementia in your care?

| Not at all | Somewhat | Extremely Involved |
| --- | --- | --- |
|  |  |  |

Please elaborate on your answer: What was the reason you were/were not involved with the decisions related to the driving of person with dementia in your care? ____________

**Mobility of person with dementia in your care**

1. What is/was their primary means of transportation:
   1. Bus, Subway
   2. Taxi or Uber
   3. Driving
   4. Having family or others driving them
   5. Walking
   6. Other, please specify: _________
2. What is/was the status of their driver’s license (after their diagnosis around the time they were still driving)?
   1. They have a valid driver’s license. Please indicate the type: ______
   2. They do not currently have a valid driver’s license as they did not renew it or they have let it expire. Please specify why?________
   3. They do not currently have a valid driver’s license as it has been revoked. Please specify why?____________
   4. N/A
3. How often do they approximately drive? *(circle response)*
   1. everyday
   2. sometimes
   3. never
4. Has their driver's license ever been revoked?
   1. No
   2. Yes, please specify why: _________________________________________
5. If they have stopped driving, when did they stopped driving? ___
6. If they have stopped driving, why did they stop driving? _____
7. Do you consider them a safe driver? *(circle response)*
   1. Yes
   2. No, please specify why: __________________________________________
8. How likely do you think it is that they could be involved in a serious car accident in the near future?
   1. Will not happen
   2. Unlikely
   3. Possible
   4. Highly likely

## Supplementary Material B. AV Familiarity Questionnaire

Driving a vehicle requires performing different **tasks** such as right and left turns, parking the car, etc.

While performing all the driving tasks, the driver has the following **responsibilities**:

- Brake and accelerate
- Steer
- Monitor the environment
- Monitor vehicle performance
- Respond in an emergency

These **driving responsibilities** are historically performed by the human driver.

In recent years, some vehicles have features that enable them to perform some of these driving responsibilities automatically. An example of these automated vehicles is ***Tesla Autopilot*** that is commercially available.

1. How familiar are you with Tesla autopilot?

| Not at all familiar | Somewhat familiar | Familiar |
| --- | --- | --- |
|  |  |  |

1. How much experience do you have with Tesla Autopilot?

| No experience | Some experience | Experienced |
| --- | --- | --- |
|  |  |  |

***If answered familiar (somewhat or extremely familiar) proceed with other questions (and mark option (a) below), otherwise end this part of the interview and move to the next part of the interview (and mark option (b) below).*

- *(a) Proceed with rest*
- *(b) End here and open C.1.PAV Educational***

1. When the Tesla Autopilot system is engaged, which of the following **driving responsibilities** would you expect is performed by the driver, which by the system, and which by both?

|  | Brake and accelerate | Steering | Monitoring the environment | Monitoring vehicle performance | Responding in an emergency |
| --- | --- | --- | --- | --- | --- |
| Driver |  |  |  |  |  |
| Automation System |  |  |  |  |  |
| Both |  |  |  |  |  |

##

## Supplementary Material C. AV Acceptance Questionnaire & Interview

**Trust**

1 To what extent do you trust ***a partially automated vehicle*** for your personal use?

| Not at all | Very little | Somewhat | To a great extent |
| --- | --- | --- | --- |
| 1 | 2 | 3 | 4 |

- What is it about partially automated vehicles that you trust/do not trust for your personal use?

2. To what extent do you trust the person with dementia in you care to drive ***a partially automated vehicle?***

| Not at all | Very little | Somewhat | To a great extent |
| --- | --- | --- | --- |
| 1 | 2 | 3 | 4 |

- What is it about partially automated vehicles that you trust/do not trust for the use of the person in your care?
- If you had different answers for yourself and the PWD in your care, why?

**Safety**

3. To what extent would you feel safe while using ***a partially automated vehicle*** for your personal use?

| Not at all | Very little | Somewhat | To a great extent |
| --- | --- | --- | --- |
| 1 | 2 | 3 | 4 |

- What is it about partially automated vehicles that you find safe/unsafe for your personal use?

4. To what extent do you think it would be safe if the person with dementia in your care drove ***a partially automated vehicle?***

| Not at all | Very little | Somewhat | To a great extent |
| --- | --- | --- | --- |
| 1 | 2 | 3 | 4 |

- What is it about partially automated vehicles that you find safe/unsafe for the use of the person in your care?
- If you had different answers for yourself and the PWD in your care, why?

**Intention to Use**

5. If ***a partially automated vehicle*** becomes available to you, how likely is it for you to use it?

| Not at all | Very little | Somewhat | To a great extent |
| --- | --- | --- | --- |
| 1 | 2 | 3 | 4 |

- What is it about partially automated vehicles that makes ***you*** want/do not want to use it?

6. If ***a partially automated vehicle*** becomes available to the person with dementia in your care, how likely is it for you to encourage them to use it?

| Not at all | Very little | Somewhat | To a great extent |
| --- | --- | --- | --- |
| 1 | 2 | 3 | 4 |

- What is it about partially automated vehicles that makes you encourage/discourage the person in your care from using it?
- If you had different answers for yourself and the PWD in your care, why?

## Supplementary Material D. AV Usefulness Questionnaire & Interview

**Part A. Driving Conditions**

**1. Clear day time**

| Do you discourage the person with dementia in your care from driving under this condition? | \| **Yes** \| **No** \| \| --- \| --- \| \|  \|  \| |
| --- | --- | --- | --- | --- | --- |
| *** proceed if answer = Yes *** | |
| Would you still discourage them from driving in this condition if they use ***a partially automated vehicle?*** | \| **Yes** \| **No** \| \| --- \| --- \| \|  \|  \| |
| Why? |  |

**2. Bad weather**

| Do you discourage the person with dementia in your care from driving under this condition? | \| **Yes** \| **No** \| \| --- \| --- \| \|  \|  \| |
| --- | --- | --- | --- | --- | --- |
| *** proceed if answer = Yes*** | |
| Would you still discourage them from driving in this condition if they use ***a partially automated vehicle?*** | \| **Yes** \| **No** \| \| --- \| --- \| \|  \|  \| |
| Why? |  |

**3. Night**

| Do you discourage the person with dementia in your care from driving under this condition? | \| **Yes** \| **No** \| \| --- \| --- \| \|  \|  \| |
| --- | --- | --- | --- | --- | --- |
| *** proceed if answer = Yes*** | |
| Would you still discourage them from driving in this condition if they use ***a partially automated vehicle?*** | \| **Yes** \| **No** \| \| --- \| --- \| \|  \|  \| |
| Why? |  |

**4. Heavy traffic**

| Do you discourage the person with dementia in your care from driving under this condition? | \| **Yes** \| **No** \| \| --- \| --- \| \|  \|  \| |
| --- | --- | --- | --- | --- | --- |
| *** proceed if answer = Yes*** | |
| Would you still discourage them from driving in this condition if they use ***a partially automated vehicle?*** | \| **Yes** \| **No** \| \| --- \| --- \| \|  \|  \| |
| Why? |  |

**5. Long distances**

| Do you discourage the person with dementia in your care from driving under this condition? | \| **Yes** \| **No** \| \| --- \| --- \| \|  \|  \| |
| --- | --- | --- | --- | --- | --- |
| *** proceed if answer = Yes*** | |
| Would you still discourage them from driving in this condition if they use ***a partially automated vehicle?*** | \| **Yes** \| **No** \| \| --- \| --- \| \|  \|  \| |
| Why? |  |

**6. Unfamiliar areas**

| Do you discourage the person with dementia in your care from driving under this condition? | \| **Yes** \| **No** \| \| --- \| --- \| \|  \|  \| |
| --- | --- | --- | --- | --- | --- |
| *** proceed if answer = Yes*** | |
| Would you still discourage them from driving in this condition if they use ***a partially automated vehicle?*** | \| **Yes** \| **No** \| \| --- \| --- \| \|  \|  \| |
| Why? |  |

**7. Highway**

| Do you discourage the person with dementia in your care from driving under this condition? | \| **Yes** \| **No** \| \| --- \| --- \| \|  \|  \| |
| --- | --- | --- | --- | --- | --- |
| *** proceed if answer = Yes*** | |
| Would you still discourage them from driving in this condition if they use ***a partially automated vehicle?*** | \| **Yes** \| **No** \| \| --- \| --- \| \|  \|  \| |
| Why? |  |

**8. Other Conditions**

- Are there any other **conditions** for which you would discourage the person with dementia in your car from driving? What are they?
- Would you still discourage them from driving in these conditions if they use ***a partially automated vehicle? Please explain.***

**Part B. Driving Tasks**

**9. Parking the car (tight spaces / parallel park)**

| Do you discourage the person with dementia in your care from driving because they have difficulties performing this task? | \| **Yes** \| **No** \| \| --- \| --- \| \|  \|  \| |
| --- | --- | --- | --- | --- | --- |
| *** proceed if answer = Yes *** | |
| Would you still discourage them if they use ***a partially automated vehicle?*** | \| **Yes** \| **No** \| \| --- \| --- \| \|  \|  \| |
| Why? |  |

**10. Backing up the car**

| Do you discourage the person with dementia in your care from driving because they have difficulties performing this task? | \| **Yes** \| **No** \| \| --- \| --- \| \|  \|  \| |
| --- | --- | --- | --- | --- | --- |
| *** proceed if answer = Yes *** | |
| Would you still discourage them if they use ***a partially automated vehicle?*** | \| **Yes** \| **No** \| \| --- \| --- \| \|  \|  \| |
| Why? |  |

**11. Taking a left turn at intersections**

| Do you discourage the person with dementia in your care from driving because they have difficulties performing this task? | \| **Yes** \| **No** \| \| --- \| --- \| \|  \|  \| |
| --- | --- | --- | --- | --- | --- |
| *** proceed if answer = Yes *** | |
| Would you still discourage them if they use ***a partially automated vehicle?*** | \| **Yes** \| **No** \| \| --- \| --- \| \|  \|  \| |
| Why? |  |

**12. Other tasks**

- Are there any other **driving** **tasks** that you would discourage the person in your care from performing? What are they?
- Would you still discourage them if they use ***a partially automated vehicle? Please explain.***
